# Endometriosis as an Uncommon Cause of Intestinal Obstruction—A Comprehensive Literature Review

**DOI:** 10.3390/jcm12196376

**Published:** 2023-10-06

**Authors:** Florentina Mușat, Dan Nicolae Păduraru, Alexandra Bolocan, Alexandru Constantinescu, Daniel Ion, Octavian Andronic

**Affiliations:** 1 General Surgery Department, Carol Davila University of Medicine and Pharmacy, The University Emergency Hospital of Bucharest, 050098 Bucharest, Romania; florentina.musat@drd.umfcd.ro (F.M.); bolocan.alexa@gmail.com (A.B.); dr.daniel.ion@gmail.com (D.I.); octavian.andronic@umfcd.ro (O.A.); 2 Gastroenterology Department, Carol Davila University of Medicine and Pharmacy, The University Emergency Hospital of Bucharest, 050098 Bucharest, Romania; alexandruconstantinescu1991@gmail.com

**Keywords:** endometriosis, intestinal, bowel, obstruction, occlusion

## Abstract

The prevalence of intestinal endometriosis has been estimated to be between 3% and 37% of all endometriosis cases. Cases of intestinal occlusion due to endometriosis foci on the small bowel and on the large bowel are even rarer, with a reported prevalence of 0.1–0.7%. The aim of this literature review was to summarize the available published evidence on the diagnosis, characteristics, and management of intestinal occlusion due to endometriosis. The search on PubMed retrieved 295 records, of which 158 were rejected following a review of the title and abstract. After reviewing the full text, 97 studies met the Population, Intervention, Comparator, Outcomes, and Study (PICOS) criteria and were included in the analysis. The total number of patients with bowel occlusion due to endometriosis included in the studies was 107. The occlusive endometrial foci were localized on the ileum in 38.3% of the cases, on the rectosigmoid in 34.5% of the cases, at the ileocecal junction and the appendix in 14.9% of the cases, and at the rectum in 10.2% of the cases. Only one case reported large bowel obstruction by endometriosis of the hepatic flexure of the colon extending to the transverse colon (0.9%), and in one case the obstruction was caused by an omental giant endometrioid cyst compressing the intestines. We identified six cases of postmenopausal females with acute bowel obstruction due to endometriosis. Malignant degeneration of endometriosis was also identified as a cause of intestinal occlusion. The mechanisms of obstruction include the presence of a mass in the lumen of the intestine or in the wall of the intestine, extrinsic compression, adhesions, or intussusception.

## 1. Introduction

Mechanical intestinal occlusions represent a surgical emergency with a varied etiology that can be encountered in any age group and represent 15% of all emergency hospitalizations presented as abdominal pain [1]. It is considered that the incidence of intestinal occlusions in the USA is 1.47 per 100,000 inhabitants [2] and that this increases with age, with an average age at diagnosis of 64 years [3]. In terms of location, the type of obstruction includes mechanical intestinal occlusions of the small intestine (small bowel obstruction, SBO) and occlusions of the large intestine (large bowel obstruction, LBO).

SBOs are responsible for approximately 300,000 admissions per year in the USA and represent between 12% and 16% of surgical ward admissions [4]. In 90% of cases, the causes of SBO are represented by adhesions, hernias, and tumors, with peritoneal adhesions being the most common etiology [5].

Occlusions of the large intestine represent approximately 25% of all intestinal occlusions [6] and 2–4% of admissions to the surgery department [7]. The most important cause of LBO is neoplasia (60% of LBO) [8]. Volvulus (10–15%) and chronic diverticular disease (10%) are the next most frequent causes of LBO. The remaining 10–15% of LBOs are due to less frequent pathologies, including Chron’s disease, bacterial or parasitic infections, and endometriosis [1,8].

Endometriosis is defined as the presence of endometrial tissue outside the uterine cavity, involving a chronic inflammatory process, and was described for the first time in 1690 [9]. Endometriosis is a condition diagnosed in 10–15% of women of reproductive age [10], most frequently in patients aged between 25 and 45 years old [11,12,13]. Although the pathogenesis of this condition has not been fully elucidated, over time three theories have been suggested to explain the abnormal localization of endometrial tissue. One theory suggests that the endometrial tissue is transplanted outside the uterine cavity either through the retrograde menstrual flow, through the blood or lymphatic flow, or during surgical interventions [12]. Another theory supports the formation of the endometrial tissue in situ from the cellular remains of the Müllerian ducts, the embryo-fetal structure from which the fallopian tubes, uterus, cervix, and part of the vagina will develop in women [12]. Finally, according to a third theory, exfoliated endometrial cells penetrate and implant in the peritoneal cavity, inducing the adjacent epithelial cells to transform into endometrial tissue [14]. After the model of cancerous cells dissemination, Roth and collaborators proposed in 1973 the theory of perineural spread of the endometrioid tissue along the autonomic nerves from the pelvis [15]. This theory was later supported by other studies that found an association between the presence of rectovaginal endometriosis and the involvement of the uterine nerve supply located in the uterosacral ligaments [16].

Endometriosis foci can be found both at the level of intraperitoneal organs (ovaries, external surface of the uterus, fallopian tubes, ligaments of the uterus, large intestine, small intestine, and appendix) and extraperitoneal ones (inguinal region, vagina, vulva, perineum, lung pleura, skin, muscle tissue, and limbs) [17]. The pathophysiology of endometriosis affecting the bowel is complex and encompasses a multifaceted interplay of factors [18]. This includes anatomical considerations, as well as processes involving invasion, fibrosis, and angiogenesis. Additionally, emerging perspectives suggest potential involvement of local neurogenesis and somatic cancer-driver mutations (e.g., in KRAS), which could potentially offer promising avenues for future therapeutic interventions. Notably, bowel endometriosis exhibits a predilection for the rectum and sigmoid colon, a phenomenon that may be linked to anatomical factors such as the deposition of refluxed endometrial tissue within the pouch of Douglas and its confinement by the sigmoid colon’s positioning. The prevalence of intestinal endometriosis is between 3% and 37% of all endometriosis cases [19]. The frequency of intestinal segments’ involvement decreases in the following order: sigmoid colon and rectum (72%), cecal appendix, terminal ileum, cecum, and transverse colon [20]. Cases of intestinal occlusion due to endometriosis foci on the small bowel and on the large bowel are even rarer, with a reported prevalence of 0.1–0.7% [21,22]. To the best of the author’s knowledge, a comprehensive synthesis of a substantial number of cases concerning intestinal occlusions caused by endometriosis has not been previously documented in existing literature. The aim of this literature review was to summarize the available published evidence on the diagnosis, characteristics, and management of intestinal occlusion due to endometriosis.

## 2. Materials and Methods

**Figure 1:**
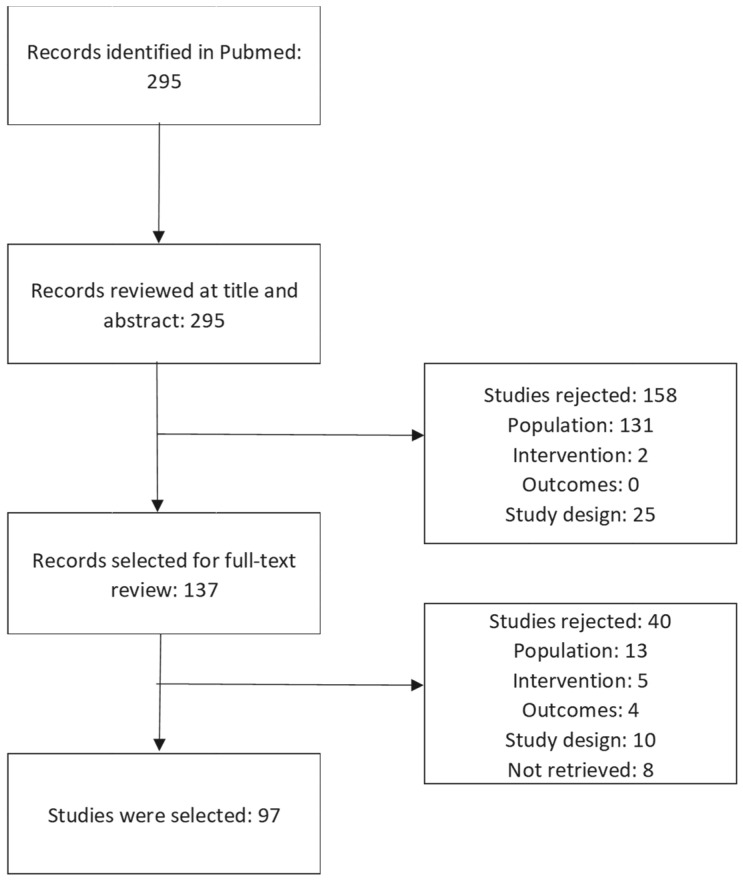
PRISMA diagram.

The methodology of the review followed the recommendations of the Cochrane Collaboration [23] and the Centre for Reviews and Dissemination [24] and relied on a set of predefined Population, Intervention, Comparator, Outcomes, and Study Design (PICOS) criteria detailed in Table 1. The search was conducted in PubMed on 16 September 2022. All articles, from database inception until 16 September 2022, have been retrieved and reviewed for inclusion based on their title and abstract by two independent reviewers, and any conflicts were resolved by a third reviewer. The search was performed using the terms “endometriosis” and “intestinal” or “bowel”, “obstruction” or “occlusion”. Search results and the number of included studies are summarized in a PRISMA [25] diagram provided in Figure 1.

Only studies that were published in English and only studies for which the full text was available online were selected. For older studies for which journal issues were not available in electronic format, the full text articles could not be retrieved (8 articles) and were excluded (Figure 1). We aimed to identify the initial treatment applied at the time of the occlusive event, the surgical procedure performed and the approach (laparoscopic or classic), the additional lesions discovered, and the role of colonoscopy in the diagnosis and management of the obstruction. Data regarding age, nationality, history of endometriosis, and history of in vitro fertilization were also collected.

Data that were not available or clearly stated were described as “not reported” and presented as a separate category. The data were synthesized and reported as mean and median, accompanied by range and standard deviation. Relevant variables extracted from the identified studies were presented descriptively in tables. All studies that were found eligible according to the PICOS criteria were included in the data description and synthesis.

## 3. Results

The systematic search based on PICOS criteria and the PRISMA statement [25] identified only case reports or case series, and no clinical trials or cohort studies. The search on PubMed retrieved 295 records, of which 158 were excluded following the review of the title and abstract. After reviewing the full text, 97 studies met the PICOS criteria and were included in the analysis. The total number of patients with bowel occlusion due to endometriosis included in the studies was 107. The complete list of studies included in the review is provided in Supplementary Table S1. The majority of the studies reported a complete description of the endometriosis cases, including diagnosis details and treatment approaches. A surgical treatment description was not reported in only two studies.

### 3.1. Studies’ Characteristics

One third of the studies (*n* = 33) were published in the last 5 years (2017–2022) and two thirds (*n* = 61) were published in the last 10 years (2012–2022). The first patient with this condition was reported in England in 1954. Most of the studies were published in Europe (*n* = 43), followed by Asia (*n* = 24), North America (*n* = 14), Australia (*n* = 7), South America (*n* = 5), and Africa (*n* = 3). Among European studies, the highest number were published in the UK (14 out of 43) and among Asian studies the highest number were published in Japan (8 out of 24).

### 3.2. Patients’ Characteristics

The average age of the patients presented in the studies was 40.3 (SD 8.97), with a median of 40 (range 22 to 78 years old). Only one patient was under 25 years old, twenty patients were over 45 years old, and five patients were over 60 years old.

Out of 107 patients, 26 were previously diagnosed with endometriosis, while the rest (81 patients) were diagnosed with endometriosis in the context of an intestinal occlusion event. In five cases, patients had a history of in vitro fertilization [21,26,27,28].

The occlusive endometrial foci were localized on the ileum in 38.3% of the cases, on the rectosigmoid in 34.5% of the cases, at the ileocecal junction and the appendix in 14.9% of the cases, and at the rectum in 10.2% of the cases. Only one case [29] reported large bowel obstruction by endometriosis of the hepatic flexure of the colon extending to the transverse colon (0.9%), and in one case [30] the obstruction was caused by an omental giant endometrioid cyst with a 45 cm diameter and weighing 4.5 kg, compressing the intestines.

Bowel obstruction due to endometriosis is a diagnosis usually made in young women of reproductive age. However, we identified six exceptions to that rule in postmenopausal females with acute bowel obstruction due to endometriosis [30,31,32,33,34,35] (Table 2).

### 3.3. General Considerations regarding Treatment Options

#### 3.3.1. Colonoscopy Utility in the Emergency Setting

For 39 patients, the diagnostic workup in the emergency room included performing a colonoscopy. In the majority of cases, colonoscopy was used only as a diagnostic tool in the emergency preoperative workup. In three cases [36,37,38], the decompression of the large bowel was achieved in the emergency setting via the placement of a metallic stent during colonoscopy. This procedure allowed the surgical treatment to be postponed until the patient recovered from the acute obstruction episode. All three cases of stent placement were performed in Europe in 2013, 2015, and 2018. In two cases, after the colonoscopy stenting, the surgical procedure was performed laparoscopically after 5 [36] and 7 days, respectively [38]. In one instance [39] a balloon dilatation was attempted as an emergency treatment for large bowel obstruction, but the procedure failed and the symptoms persisted. Apart from the three cases already mentioned, another case of metallic stent placement [29] was described in one patient with hepatic flexure of the colon endometrioses. In that case, following consultations with specialists in gynecology and colorectal surgery, the patient opted for a conservative therapeutic approach involving the administration of intramuscular leuprolide. However, one week subsequent to this intervention, she experienced a recurrence of symptoms and was subsequently rehospitalized. Computed tomography imaging revealed ongoing obstruction at the hepatic flexure of the colon. As a therapeutic measure to alleviate the large bowel obstruction and facilitate additional time for evaluating the response to leuprolide treatment, while planning for potential surgical intervention, a colonic stent was inserted. Since the endometrioid mass did not respond to the leuprolide treatment and there was a risk of bowel perforation at the stent location, the surgical team performed a total colectomy.

#### 3.3.2. Conservative Treatment in the Emergency Setting

Conservative treatment performed more than 24 h before surgery was reported in 15 patients and referred to bowel rest, a naso-enteric tube, antibiotics, fluid resuscitation, or hormonal pharmacological treatment targeting endometriosis in patients with biopsy confirmation.

#### 3.3.3. Surgical Treatment in the Emergency Setting

The surgical interventions were laparotomies or laparoscopies in 86 and in 20 cases, respectively (Table 3 and Table 4). Three laparoscopic interventions were converted to laparotomies and two were performed as single-incision laparoscopic surgery (SILS). In one case [29], that of the patient who was diagnosed with endometriosis of the hepatic flexure of the colon, the patient did not undergo surgery because conservative treatment with intramuscular leuprolide was initiated after the biopsy, along with endoscopic placement of a metallic stent. In another instance of rectosigmoid endometriosis [37], there was no need for surgical treatment to solve the bowel occlusion since a metallic stent was placed endoscopically.

The laparoscopic interventions for occlusive bowel endometrioses were performed between 2015 and 2022 (18 interventions), with the exception of one intervention successfully performed in 2001 in Japan, and another one that took place in Greece in 2007 but was converted to laparotomy. Seven laparoscopies were performed in Asian countries, seven in Europe, three in Australia, and three in North America. The laparoscopic interventions were used when the site of the occlusion was located on the ileum (eleven patients), ileocecal (two patients), the sigmoid colon (two patients), the rectosigmoid junction (two patients), the rectum (two patients), and the cecal appendix (one patient).

Although the intraoperative abdominal inspection can reveal multiple adenopathies, most of them are reactive. Even so, in 16 out of 107 patients, the histopathological examination revealed lymph node involvement by endometrial foci. This fact can support the theory of lymphatic spread of the endometrial tissue out of the uterine cavity.

### 3.4. Malignant Degeneration of Endometriosis

Although endometriosis is considered a benign cause of intestinal obstruction, the malignant degeneration of this disease was reported in two cases of endometrioid adenocarcinoma of the ileum in a 45-year-old woman [51] and the sigmoid colon in a 78-year-old woman [33]. Both patients had a history of genital endometriosis and received hormone replacement therapy with estrogens after they underwent bilateral oophorectomy, total abdominal hysterectomy, and salpingooophorectomy. In another study, the authors described the particular case of a 50-year-old woman, with no significant medical history, who was diagnosed with LBO due to a rectal mass [96]. The histopathological examination of the mass revealed two glandular components: colonic adenocarcinoma of the classic type, associated with transmural endometriosis implants. This case demonstrates the possibility of the simultaneous presence of endometriosis foci and other types of neoplasia in the same tumoral mass.

### 3.5. Treatment Particularities Based on the Site of Obstruction

The management of intestinal occlusion cases due to endometriosis is presented below based on the localization of the obstruction: ileal, ileocecal, rectal, sigmoid colon, and rectosigmoid.

#### 3.5.1. Ileal Obstruction

The mean age of patients diagnosed with ileal occlusions (41 cases) [26,34,35,39,40,41,42,43,44,45,46,47,48,49,50,51,52,53,54,55,56,57,58,59,60,61,62,63,64,65,66,67,68,69,70,71,72,73] was 38.71, with a minimum of 22 and a maximum of 54. Conservative treatment of ileal obstruction was first attempted in seven patients [26,34,40,53,64,67,72], five of whom were subsequently managed after a few days with laparoscopic surgery. Two of those interventions were single-incision laparoscopic surgeries, both of which were performed in Japan in 2021 and 2015 [34,40]. In one case [39], a colonoscopic balloon dilatation of the ileal stricture was attempted twice, as the authors considered Chron’s disease and tuberculosis as differential diagnosis; however, the symptomatology relapsed requiring a diagnostic laparoscopy, followed by a laparoscopic right hemicolectomy. The rest of the thirty-three cases of ileal obstruction underwent emergency surgery, six of which were approached laparoscopically. The procedures performed consisted of six ileocecal resections, twelve right hemicolectomies, nineteen ileal resections, one ileo-transverso-stomy, and one biopsy with side-to-side isoperistaltic ileo-transverso-anastomosis. Lymph node involvement was encountered in the histopathological results of seven cases [40,42,49,51,54,55,63]. Other associated lesions were described on the right ovary, appendix, cecum, rectum, and ascending colon.

#### 3.5.2. Ileocecal Obstruction

The mean age of patients diagnosed with ileocecal occlusions (15 cases) [74,75,76,77,78,79,80,81,82,83,84,85,86,87,88] was 37.86, with a minimum of 32 and a maximum of 46. Ileocecal occlusions were initially managed conservatively in four instances, two of which were followed by two laparoscopic procedures. The procedures performed consisted of ten right hemicolectomies and five ileocecal resections. In the majority of the cases, the lesion was initially suspected to be malignant, leading to the execution of a right hemicolectomy, during which the enlarged lymph nodes were encompassed within the surgical specimen. Endometrial cells were discovered in the lymph nodes of one patient [79]. Ovarian endometriosis was described in four patients. In one patient [120], the bowel obstruction was caused by a band of fibrosis extending from the appendix to the distal ileum. The presence of endometrial foci in the appendix can lead to local inflammation, resulting in fibrosis and the formation of adhesions, which can remain asymptomatic or can determine consecutive bowel obstruction. Moreover, periodic menstrual bleeding in the ectopic tissue may trigger acute appendicitis. The treatment consisted of appendicectomy and adhesiolysis, and the histopathological exams revealed multiple endometrial nodules in the wall of the appendix.

#### 3.5.3. Rectal Obstruction

The mean age of patients diagnosed with LBO due to rectal endometriosis (11 patients) [27,89,90,91,92,93,94,95,96,97] was 38.45, with a minimum of 26 and a maximum of 50. Nine underwent emergency surgery, consisting of a total colectomy in one patient, a colostomy without any type of resection in three patients, anterior rectal resection in three patients, and rectosigmoid resection in four patients. In the context of emergency surgical interventions, it may be imperative to consider the implementation of a prophylactic ileostomy as part of the operative strategy. This consideration arises because of the presence of intestinal edema and the attendant risk of postoperative anastomotic fistula formation. Rectal endometriosis was associated with extensive lesions in the uterus and ovaries, named ‘‘the frozen pelvis’’ by some authors. In those situations, the surgical intervention involved total hysterectomy to provide access to the rectum. Lymph node involvement was described in two cases [89,97].

#### 3.5.4. Sigmoid Colon Obstruction

The mean age of patients diagnosed with LBO due to sigmoid colon endometriosis (14 patients) [28,32,33,36,98,99,100,101,102,103,104,105,106,107] was 43, with a minimum of 31 and a maximum of 78. In the majority of cases, the initial treatment consisted of an emergency surgery (12 patients) with the exception of two cases where a therapeutic colonoscopy was first attempted. Seven Hartman procedures, one hemicolectomy with colostomy, five sigmoid colectomies with primary anastomosis, and one sigmoid colostomy were reported. LBO arising from sigmoid colon endometriosis significantly impacts patients’ quality of life. Notably, surgical interventions resulted in colostomy formation in nine of these cases, further underscoring the profound implications of this condition on patients’ well-being. The endometrial lesion on the sigmoid was associated with lymph node involvement (three patients) [104,105,106] and left ovary endometriosis (two patients).

#### 3.5.5. Rectosigmoid Obstruction

The mean age of patients diagnosed with LBO due to rectosigmoidian endometriosis (23 patients) [21,31,37,38,108,109,110,111,112,113,114,115,116,117,118,119] was 40.47, with a minimum of 25 and a maximum of 63. Surgical intervention was delayed by the conservative treatment in only one patient, and in other two patients a stent was placed by colonoscopy. The interventions referred to six Hartman procedures, eight colostomies without resection, five rectosigmoid resections with anastomosis, and three anterior resections with anastomosis. The approach was laparoscopic in two cases of rectosigmoid resection with anastomosis. In a case series documented by de Jong et al. [111], patient management required the placement of ureteral stents. This intervention became imperative because patients presented with initial symptoms or indications of unilateral or bilateral hydronephrosis, coupled with a progressive decline in renal function. These clinical manifestations were attributed to the obstruction of the distal ureter, primarily caused by retroperitoneal endometriosis and/or the presence of fibrotic tissue. In the same study, the biopsy conducted during the supplementary rectosigmoidoscopy did not provide significant diagnostic assistance. Specifically, in one case, examination of a biopsy specimen extracted from the stenotic lesion revealed features consistent with non-specific colitis, devoid of any malignancy indicators, with the endoscopic observation of a normal mucosal appearance. Lymph nodes were involved in endometriosis in three cases [109,110,119] and additional intrabdominal endometrial foci were described in four other cases. 

## 4. Discussion

The treatment dedicated to intestinal occlusions is traditionally surgical. Technological advances currently allow, in selected cases, the postponement of surgical intervention and the resolution of the occlusive phenomenon by means of various colonoscopic maneuvers, such as balloon dilatation or intraluminal metallic stent placement [121].

The studies carried out on inflammatory bowel disease or anastomotic stenoses showed that balloon dilatation is more efficient in the case of benign lesions than in the case of malignant occlusions [122]. Occlusion cases are selected for balloon dilation based on specific criteria [123]. For example, stenoses narrower than 10 mm and with a length of less than 4 cm are more likely to be successfully treated by this procedure. In addition, cases with urgent surgical indications, such as digestive perforation, represent the only absolute contraindication [123]. The alleviation of obstruction through balloon dilatation serves several pivotal purposes in the clinical management of such cases. Firstly, it enables the correction of fluid and electrolyte imbalances, thereby stabilizing the patient’s overall condition. Secondly, it facilitates the administration of a comprehensive bowel preparation, a vital preparatory step in surgical interventions. Lastly, this maneuver enables the possibility of conducting a single-stage resection and anastomosis during surgery. Essentially, this procedure affords the surgical team an extended timeframe, effectively “extending the temporal window”, to optimize patient outcomes. The disadvantage of balloon dilatation in benign stenoses is that, often, this procedure must be repeated to ensure the patency of the intestinal lumen [122].

Self-expanding stents, dedicated specifically to neoplasia, have a high success rate in the case of benign stenoses (95%) but are associated with a high percentage of complications, especially after the seventh day post-installation [124]. The installation of self-expandable stents is contraindicated in lesions of the lower rectum as it may cause tenesmus and incontinence [125]. Although the ideal moment for the extraction of the stent is not clearly established, it is considered that the stent should be removed 4–8 weeks after the procedure to prevent tissue embedding [126].

There are no studies investigating the effectiveness of the two types of colonoscopic procedures in intestinal occlusions due to endometriosis. However, colonoscopic treatment allows for the visualization of the type of lesion (protrusive mass or intramural lesion) and for the confirmation of diagnosis through biopsy. Targeted treatment for endometriosis can be administered based on the histopathological diagnosis. Targeted treatment options include oral contraceptives based on estrogen–progesterone, levonorgestrel intrauterine devices or oral gestagens, gonadotropin-releasing hormone (GnRH) agonists with or without add-back therapy, aromatase inhibitors, and danazol [127]. Conservative management is of particular importance in lower rectal endometriosis, considering that surgical interventions in this intensely vascularized and innervated anatomical region are associated with increased morbidity [128].

Early studies did not show a significant response to pharmacological therapy in patients with endometriosis of the digestive tract [129]. However, recent studies [130,131] have shown the effectiveness of oral contraceptives, levonorgestrel intrauterine devices, and oral gestagens in the case of patients with recto-sigmoid endometriosis. However, it is worth mentioning that the patients included in these studies were diagnosed with nonocclusive recto-sigmoid endometriosis.

One interesting observation is that patients diagnosed at a young age (under 30 years) with endometriosis of the digestive tract present a more aggressive form of the disease and a more limited response to conservative treatment than older patients [131,132].

Out of the 107 cases identified in this review, colonoscopic management of the obstruction was attempted in five cases. In four cases, this procedure only served as a bridge to surgery. Only one patient received pharmacological treatment after the insertion of a self-expandable stent.

Therefore, intestinal occlusions determined by the presence of intraluminal or intramural endometriosis nodules could initially be managed conservatively. First, a colonoscopy should be performed to determine the location and appearance of the lesion and to take biopsies. After the remission of the occlusive symptoms by endoscopic balloon dilatation or stenting, pharmacological treatment for endometriosis can be initiated. The response to the pharmacological treatment must be evaluated clinically and by MRI, in order to establish the need for a subsequent surgical procedure or to continue the conservative treatment. 

In practice, procedures such as colonoscopy, balloon dilatation, or insertion of an auto-expandable stent are not always available in the emergency setting. Nevertheless, even when all the technical conditions are met, the success of endoscopic management is not guaranteed. According to the analyzed data, the treatment of intestinal occlusion due to endometriosis is mainly surgical, and most of the interventions are performed through a midline laparotomy. There are authors citing attempts of endoscopic treatment, but this type of management is not standardized for endometrial disease. Considering that 75% of patients were diagnosed with endometriosis at the time of emergency presentation for occlusive symptoms, establishing the diagnosis and a targeted therapeutic plan is challenging.

Although Pubmed-MEDLINE is a comprehensive database, some relevant papers that are not indexed in this database might have been omitted from this review. Additional bias was introduced by selecting only studies that were published in English and only studies for which the full text was available online. The literature searches identified only case reports and case series, and no metanalysis or other statistical data synthesis have been published.

## 5. Conclusions

Intestinal obstruction due to endometriosis is a rare condition, but it should be kept in mind in relation to female patients of reproductive age presenting to the emergency room with signs and symptoms of ileus. Even more rare is the occurrence of this disease in postmenopausal women, but it remains a possible diagnosis. Intraoperatively, the macroscopic appearance of the lesions may considerably resemble that of a neoplastic disease, from the aspect of the obstructive mass to the presence of lymph node involvement. This is why many procedures consist in extensive bowel resections. In other exceptional cases, benign endometriosis can transform into endometrioid adenocarcinoma. The mechanism of obstruction refers to the presence of a mass in the lumen of the intestine or in the wall of the intestine, extrinsic compression, adhesions, or intussusception.

Colonoscopic treatment in the emergency setting can provide the necessary framework for pharmacological treatment of endometriosis initiation. Being a rare condition, there are only case reports or case series reporting the clinical setting, the management, and the outcomes.

## Figures and Tables

**Table 1:** PICOS criteria.

Criteria	Inclusion Criteria	Exclusion Criteria
Population	Female diagnosed with acute intestinal occlusion by endometriosis, not pregnant	Other causes of intestinal occlusion
Intervention	Any cases treated surgically	Cases treated non-surgically
Outcomes	Patient characteristics Treatment for intestinal occlusion Complications Survival	Studies not reporting any of the outcomes of interest
Study Design	Case reports Prospective observational studies Case-control studies Retrospective studies Database analyses Controlled studies Randomized controlled studies Non-randomized controlled studies	Review Letter Note Comment Editorial Systematic reviews (kept for cross-checking at full text review stage)

**Table 2:** Cases of acute bowel obstruction caused by endometriosis in postmenopausal women. LT—laparotomy; SILS—single excision laparoscopic surgery.

Author, Year	Country	Patient Age	Previously Diagnosed Endometriosis	Site of the Obstruction	Initial Treatment	Surgical Procedure	Surgical Approach
Bidarmaghz et al. 2016 [31]	Australia	63	No	Rectosigmoid	Surgical	Hartmann’s procedure	LT
Deval et al. 2002 [32]	France	63	No	Sigmoid colon	Surgical	Hartmann’s procedure	LT
Wang et al. 2011 [33]	UK	78	Yes	Sigmoid colon	Surgical	Hartmann’s procedure	LT
Naem et al. 2020 [30]	Syria	67	No	Omental giant cyst	Surgical	Cystectomy and omentectomy	LT
Izuishi et al. 2015 [34]	Japan	54	No	Ileum	Conservative	Partial resection of the small bowel with end-to-end anastomosis	SILS
Popoutchi et al. 2008 [35]	Brazil	74	No	Ileum	Surgical	Segmental enterectomy	LT

**Table 3:** Localization of the occlusive endometrial foci and the management performed in the emergency setting. LT—laparotomy; LS—laparoscopy; no—number. * Proportion of patients presenting with various types of occlusion calculated out of the total number of cases identified in the review (*n* = 107).

				Initial Treatment			Surgical Treatment	
Site of the Occlusive Endometrial Foci	Number of Patients	% *	Mean Age	Conservative (for More than 24 h)	Emergency Surgery	Therapeutic Colonoscopy	LT	LS
Ileal [26,34,35,39,40,41,42,43,44,45,46,47,48,49,50,51,52,53,54,55,56,57,58,59,60,61,62,63,64,65,66,67,68,69,70,71,72,73]	41	38.3	42.06	7	33	1	30	11
Ileocecal [74,75,76,77,78,79,80,81,82,83,84]	11	10.2	37.55	4	7	0	9	2
Cecal [85,86,87,88]	4	3.7	38.75	0	4	0	4	0
Rectal [27,89,90,91,92,93,94,95,96,97]	11	10.2	38.45	2	9	0	9	2
Sigmoidian [28,32,33,36,98,99,100,101,102,103,104,105,106,107]	14	13	43.00	0	12	2	12	2
Rectosigmoidian [21,31,37,38,108,109,110,111,112,113,114,115,116,117,118,119]	23	21.5	40.48	1	20	2	20	2
Hepatic Flexure of the Colon [29]	1	0.9	39	1	0	0	1	0
Appendiceal [120]	1	0.9	29	0	1	0	0	1
Others (Omental Giant Cyst) [30]	1	0.9	67	0	1	0	1	0
Total	107	100	-	15	87	5	86	20

**Table 4:** Surgical management of the endometrial bowel obstruction. LS—laparoscopy; LT—laparotomy; N.R.—not reported. * The table does not include 3 patients with endometriosis of hepatic flexure of the colon, of the appendix, and of the omentum).

			Digestive Continuity (Number of Patients)			Approach (Number of Patients)	
Site of the Occlusive Endometrial Foci	Procedure	Total	Anastomosis	Stoma	N.R.	LS	LT
Ileal	Ileocecal resection	6	2	1	3	2	4
	Right hemicolectomy	12	7		5	6	6
	Ileal resection	19	11	1	7	3	16
	Ileo-transverso-stomy	1	-	-	-	0	1
	Biopsy and side-to-side isoperistaltic ileo-transverso-anastomosis	1	-	-	-	0	1
	N.R.	2	-	-	-	-	-
Ileocecal	Right hemicolectomy	8	6	1	1	1	7
	Ileocecal resection	3	1	1	1	1	2
Cecal	Right hemicolectomy	2	2	0	-	0	2
	Ileocecal resection	2	1	0	1	0	2
Rectal	Total colectomy	1	0	1	0	0	1
	Colostomy	3	0	3	0	0	3
	Anterior resection	3	2	0	1	1	2
	Rectosigmoid resection	4	0	1	3	1	3
Sigmoidian	Hartman procedure	7	0	7	0	0	7
	Left hemicolectomy	1	0	1	0	0	1
	Sigmoid colectomy	5	5	0	0	2	3
	Sigmoid colostomy	1	0	1	0	0	1
Rectosigmoidian	Hartman procedure	6	0	6	0	0	6
	Colostomy	8	0	8	0	0	8
	Rectosigmoid resection	5	5	0	0	2	3
	Anterior resection No surgery	3 1	3 0	0 0	0 0	0 0	3 0
Total Number of Patients *		104	45	32	22	19	82

## Data Availability

Full data set can be available upon request. The review was not registered in a database and the protocol is not publicly available.

## Supplementary Materials

The following supporting information can be downloaded at: https://www.mdpi.com/article/10.3390/jcm12196376/s1, Table S1: Selected articles and the number of cases included from each study.

## References

[1] Cappell M.S., Batke M. (2008). Mechanical Obstruction of the Small Bowel and Colon. Med. Clin. N. Am..

[2] Hill A.G. (2008). The Management of Adhesive Small Bowel Obstruction—An Update. Int. J. Surg..

[3] Drożdż W., Budzyński P. (2012). Change in mechanical bowel obstruction demographic and etiological patterns during the past century: Observations from one health care institution. Arch. Surg..

[4] Maung A.A., Johnson D.C., Piper G.L., Barbosa R.R., Rowell S.E., Bokhari F., Collins J.N., Gordon J.R., Ra J.H., Kerwin A.J. (2012). Evaluation and Management of Small-Bowel Obstruction: An Eastern Association for the Surgery of Trauma Practice Management Guideline. J. Trauma Acute Care Surg..

[5] Richard P.G., Issa Y., van Santbrink E.J.P., Bouvy N.D., Kruitwagen R.F.P.M., Jeekel J., Bakkum E.A., Rovers M.M., van Goor H. (2013). Burden of Adhesions in Abdominal and Pelvic Surgery: Systematic Review and Met-Analysis. BMJ.

[6] Markogiannakis H., Messaris E., Dardamanis D., Pararas N., Tzertzemelis D., Giannopoulos P., Larentzakis A., Lagoudianakis E., Manouras A., Bramis I. (2007). Acute Mechanical Bowel Obstruction: Clinical Presentation, Etiology, Management and Outcome Rapid Communication. World J. Gastroenterol..

[7] Taourel P., Kessler N., Lesnik A., Pujol J., Morcos L., Bruel J.M. (2003). Helical CT of Large Bowel Obstruction. Abdom. Imaging.

[8] Catena F., de Simone B., Coccolini F., di Saverio S., Sartelli M., Ansaloni L. (2019). Bowel Obstruction: A Narrative Review for All Physicians. World J. Emerg. Surg..

[9] Benagiano G., Brosens I., Lippi D. (2014). The History of Endometriosis. Gynecol. Obstet. Investig..

[10] Zondervan K.T., Becker C.M., Missmer S.A. (2020). Endometriosis. N. Engl. J. Med..

[11] Maggiore U.L.R., Ferrero S., Mangili G., Bergamini A., Inversetti A., Giorgione V., Viganò P., Candiani M. (2016). A Systematic Review on Endometriosis during Pregnancy: Diagnosis, Misdiagnosis, Complications and Outcomes. Hum. Reprod. Update.

[12] Smolarz B., Szyłło K., Romanowicz H. (2021). Endometriosis: Epidemiology, Classification, Pathogenesis, Treatment and Genetics (Review of Literature). Int. J. Mol. Sci..

[13] Eskenazi B., Warner M.L. (1997). Epidemiology Of Endometriosis. Obstet. Gynecol. Clin. N. Am..

[14] Nap A.W., Groothuis P.G., Demir A.Y., Evers J.L.H., Dunselman G.A.J. (2004). Pathogenesis of Endometriosis. Best Pract. Res. Clin. Obstet. Gynaecol..

[15] Roth L.M. (1973). Endometriosis with Perineural Involvement. Am. J. Clin. Pathol..

[16] Possover M., Rhiem K., Chiantera V. (2005). The “Neurologic Hypothesis”: A New Concept in the Pathogenesis of the Endometriosis?. Gynecol. Surg..

[17] Ion D., Bolocan A., Piţuni S.M., Mateoiu P.E., Musat F., Andronic O., Pǎduraru D.N. (2017). Concomitant Inguinal Endometriosis and Groin Hernia—Case Report. Arch. Balk. Med. Union.

[18] Yong P.J., Bedaiwy M.A., Alotaibi F., Anglesio M.S. (2021). Pathogenesis of bowel endometriosis. Best Pract. Res. Clin. Obstet. Gynaecol..

[19] Sánchez-Cifuentes Á., Candel-Arenas M.F., Albarracín-Marín-Blázquez A. (2016). Intestinal Endometriosis. Our Experience. Rev. Esp. Enferm. Dig..

[20] Veeraswamy A., Lewis M., Mann A., Kotikela S., Hajhosseini B., Nezhat C. (2010). Extragenital Endometriosis. Clin. Obstet. Gynecol..

[21] Allan Z. (2018). A Case of Endometriosis Causing Acute Large Bowel Obstruction. Int. J. Surg. Case Rep..

[22] Palcău C., Bolocan A., Luiza Nechita S., Ion D., Păduraru D.N. (2021). Acute Large Bowel Obstruction-A Rare Presentation Of Colonic Endometriosis Case Report. Rom. J. Emerg. Surg..

[23] Higgins J.P.T., Altman D.G., Gøtzsche P.C., Jüni P., Moher D., Oxman A.D., Savović J., Schulz K.F., Weeks L., Sterne J.A.C. (2011). The Cochrane Collaboration’s Tool for Assessing Risk of Bias in Randomised Trials. BMJ.

[24] Dignen B. (2008). English for Presentations.

[25] Page M.J., McKenzie J.E., Bossuyt P.M., Boutron I., Hoffmann T.C., Mulrow C.D., Shamseer L., Tetzlaff J.M., Akl E.A., Brennan S.E. (2021). The PRISMA 2020 Statement: An Updated Guideline for Reporting Systematic Reviews. BMJ.

[26] Santos-Manzur A., Valdez-Bocanegra D.R., Ornelas-Flores M.C., Pineda-Díaz J., Stoopen-Margain E. (2020). Ileal Obstruction Caused by Transmural Endometriosis in a Patient with Simultaneous C. Difficile Colitis and Influenza AH1N1. Case Report. Int. J. Surg. Case Rep..

[27] Nagakari K., Azuma D., Takehara K., Ohuchi M., Ishizaki Y., Sakamoto K. (2022). Laparoscopic Triple Segmental Bowel Resection for Endometriosis Revealed by Rectal Obstruction during Infertility Treatment. Case Rep. Gastroenterol..

[28] Quicray M., Darwish B., Bridoux V., Roman H. (2016). Bowel Occlusion in an Infertile Woman with Documented Deep Endometriosis of the Sigmoid Colon: Why Was It Not Unexpected?. Gynecol. Obstet. Fertil..

[29] Moktan V.P., Koop A.H., Olson M.T., Lewis M.D., Gomez V., Farraye F.A. (2021). An Unusual Cause of Large Bowel Obstruction in a Patient With Ulcerative Colitis. ACG Case Rep. J..

[30] Naem A., Shamandi A., Al-Shiekh A., Alsaid B. (2020). Free Large Sized Intra-Abdominal Endometrioma in a Postmenopausal Woman: A Case Report. BMC Women’s Health.

[31] Bidarmaghz B., Shekhar A., Hendahewa R. (2016). Sigmoid Endometriosis in a Post-Menopausal Woman Leading to Acute Large Bowel Obstruction: A Case Report. Int. J. Surg. Case Rep..

[32] Deval B., Rafii A., Dachez M.F., Kermanash R., Levardon M. (2002). Sigmoid Endometriosis in a Postmenopausal Woman. Am. J. Obstet. Gynecol..

[33] Wang T.T., Jabbour R.J., Girling J.C., McDonald P.J. (2011). Extraluminal Bowel Obstruction by Endometrioid Adenocarcinoma 34 Years Post-Hysterectomy: Risks of Unopposed Oestrogen Therapy. J. R. Soc. Med..

[34] Izuishi K., Sano T., Shiota A., Mori H., Ebara K. (2015). Small Bowel Obstruction Caused by Endometriosis in a Postmenopausal Woman. Asian J. Endosc. Surg..

[35] Popoutchi P., dos Reis Lemos C.R., Silva J.C., Nogueira A.A., Feres O., Ribeiro da Rocha J.J. (2008). Postmenopausal Intestinal Obstructive Endometriosis: Case Report and Review of the Literature. Sao Paulo Med. J..

[36] Calcagno P., Viti M., Cornelli A., Galli D., D’Urbano C. (2018). Intestinal Obstruction Caused by Endometriosis: Endoscopic Stenting and Expedited Laparoscopic Resection Avoiding Stoma. A Case Report and Review of the Literature. Int. J. Surg. Case Rep..

[37] Whelton C., Bhowmick A. (2013). Acute Endometrial Bowel Obstruction—A Rare Indication for Colonic Stenting. Int. J. Surg. Case Rep..

[38] Navajas-Laboa M., Orive-Calzada A., Landaluce A., Zabalza-Estevez I., Larena J.A., Arévalo-Serna J.A., Bridet L., López-López M., Torres-Burgos S., Bernal-Martínez A. (2015). Colonic Obstruction Caused by Endometriosis Solved with a Colonic Stent as a Bridge to Surgery. Arab. J. Gastroenterol..

[39] Sali P.A., Yadav K.S., Desai G.S., Bhole B.P., George A., Parikh S.S., Mehta H.S. (2016). Small Bowel Obstruction Due to an Endometriotic Ileal Stricture with Associated Appendiceal Endometriosis: A Case Report and Systematic Review of the Literature. Int. J. Surg. Case Rep..

[40] Koyama R., Aiyama T., Yokoyama R., Nakano S. (2021). Small Bowel Obstruction Caused by Ileal Endometriosis with Appendiceal and Lymph Node Involvement Treated with Single-Incision Laparoscopic Surgery: A Case Report and Review of the Literature. Am. J. Case Rep..

[41] Torralba-Morón A., Urbanowicz M., Andres C.I.-D., Lopez-Alonso G., Colina-Ruizdelgado F., Guerra-Vales J.-M. (2016). Acute Small Bowel Obstruction and Small Bowel Perforation as a Clinical Debut of Intestinal Endometriosis: A Report of Four Cases and Review of the Literature. Intern. Med..

[42] de Ceglie A., Bilardi C., Blanchi S., Picasso M., di Muzio M., Trimarchi A., Conio M. (2008). Acute Small Bowel Obstruction Caused by Endometriosis: A Case Report and Review of the Literature. World J. Gastroenterol..

[43] Lam K., Lang E. (2020). Endometriosis as a Rare Cause of Small Bowel Obstruction. ANZ J. Surg..

[44] Kobayashi K., Yamadera M., Takeo H., Murayama M. (2022). Small Bowel Obstruction Caused by Appendiceal and Ileal Endometriosis: A Case Report. J. Surg. Case Rep..

[45] Chan D.L., Chua D., Ravindran P., Perez Cerdeira M., Mor I. (2017). A Case Report of Endometriosis Presenting as an Acute Small Bowel Obstruction. Int. J. Surg. Case Rep..

[46] López Carrasco A., Hernández Gutiérrez A., Hidalgo Gutiérrez P.A., Rodríguez González R., Marijuán Martín J.L., Zapardiel I., de Santiago García J. (2017). Ileocecal Endometriosis: Diagnosis and Management. Taiwan J. Obstet. Gynecol..

[47] Zepeda M.R., Win S.K. (2021). A Rare Case of Endometriosis of the Small Bowel. Case Rep. Pathol..

[48] Morales-Morales C.A., Morales-Flores L.F., Gonzalez-Urquijo M., Suárez-Márquez E., Zambrano-Lara M., Baca-Arzaga A.A., Tijerina-Gómez L.O. (2021). Ileocolonic Intussusception Due to Severe Endometriosis. Clin. J. Gastroenterol..

[49] Arata R., Takakura Y., Ikeda S., Itamoto T. (2019). A Case of Ileus Caused by Ileal Endometriosis with Lymph Node Involvement. Int. J. Surg. Case Rep..

[50] Ávila Vergara M.A., Sánchez Carrillo V., Peraza Garay F. (2018). Bowel Obstruction Secondary to Deep Infiltrating Endometriosis of the Ileum. Rev. Esp. Enferm. Dig..

[51] Marchena-Gomez J., Conde-Martel A., Hemmersbach-Miller M., Alonso-Fernandez A. (2006). Metachronic Malignant Transformation of Small Bowel and Rectal Endometriosis in the Same Patient. World J. Surg. Oncol..

[52] Arer I.M., Yabanoglu H., Hasbay B. (2016). Anastomotic Leakage in a Patient with Acute Intestinal Obstruction Secondary to Appendiceal and Ileal Endometriosis: A Case Report. J. Clin. Diagn. Res..

[53] Khwaja S.A., Zakaria R., Carneiro H.A., Khwaja H.A. (2012). Endometriosis: A Rare Cause of Small Bowel Obstruction. BMJ Case Rep..

[54] Dhannoon A., Bajwa A., Kunna M., Canney A., Nugent E. (2022). Beyond Borders: A Case Report of Small Bowel Obstruction Secondary to Undiagnosed Florid Endometriosis. Int. J. Surg. Case Rep..

[55] Karaman K., Pala E.E., Bayol U., Akman O., Olmez M., Unluoglu S., Ozturk S. (2012). Endometriosis of the Terminal Ileum: A Diagnostic Dilemma. Case Rep. Pathol..

[56] Bratu D., Chicea R., Ciprian T., Beli L., Dan S., Mihetiu A., Adrian B. (2016). A Rare Case of Ileus Caused by Ileum Endometriosis. Int. J. Surg. Case Rep..

[57] Lea R., Whorwell P.J. (2003). Irritable Bowel Syndrome or Endometriosis, or Both?. Eur. J. Gastroenterol. Hepatol..

[58] Shah M., Tager D., Feller E. (1995). Intestinal endometriosis masquerading as common digestive disorders. Arch. Intern. Med..

[59] Ranaweera R.K., Gamage S.M., Ubayawansa D.H., Kumara M.M. (2016). Terminal Ilial Intussusception in an Adult Due to Endometriosis. BMC Res. Notes.

[60] Laiz Díez B., García Muñoz-Najar A., Durán Poveda M. (2019). Laparoscopic Management of a Small Bowel Obstruction Caused by an Endometriotic Focus. Rev. Esp. Enferm. Dig..

[61] Dong C., Ngu W.S., Wakefield S.E. (2015). Endometriosis Masquerading as Crohn’s Disease in a Patient with Acute Small Bowel Obstruction. BMJ Case Rep..

[62] Upreti S., Bansal R., Upreti S., Mathur S. (2013). Stromal Endometriosis of the Intestine: An Elusive Presentation with a Review of the Literature: A Case Report. J. Clin. Diagn. Res..

[63] Ridha J.R., Cassaro S. (2003). Acute Small Bowel Obstruction Secondary to Ileal Endometriosis: Report of a Case. Surg. Today.

[64] Poole R.W. (1961). Acute Small Bowel Obstruction Due to Endometriosis. Can. Med. Assoc. J..

[65] Gregorić P., Doklestić K., Pandurović M., Radenković D., Karadžić B., Raspopović M., Micev M., Ivančević N., Šijački A., Bajec D. (2012). Distal Ileal Endometriosis as a Cause of Ileus: A Case Report. Srp. Arh. Celok. Lek..

[66] Attar A., Lagorce C. (2007). Small Bowel Obstruction Caused by Endometriosis. Clin. Gastroenterol. Hepatol..

[67] Koutsourelakis I., Markakis H., Koulas S., Mparmpantonakis N., Perraki E., Christodoulou K. (2007). Ileocolic Intussusception Due to Endometriosis. J. Soc. Laparosc. Robot. Surg..

[68] Rancaño R.S., Choho K.K., Morales M.D., Bueno V.M.S., Soto M.d.M.D.E., Santacruz Y.R., Guillén R.A. (2022). Ileal Obstruction Due to Endometriosis, with Associated Appendiceal Involvement: A Unique and Elusive Situation. Gastroenterol. Hepatol..

[69] Fernández-Rey C.L., Álvarez-González S.A., Díaz-Solís P., Blanco-González A., Costilla-García S. (2009). Small Bowel Obstruction Secondary to Ileal Endometriosis: Multisection Computer Tomography Evaluation. Rev. Esp. Enferm. Dig..

[70] Harty R.F., Kaude J.V. (1983). Invasive Endometriosis of the Terminal Ileum: A Cause of Small Bowel Obstruction of Obscure Origin. South. Med. J..

[71] Collins P.G. (1957). Endometriosis as a Cause of Intestinal Obstruction: A Report of Two Cases. Postgrad. Med. J..

[72] Mussa F.F., Younes Z., Tihan T., Lacy B.E. (2001). Anasarca and Small Bowel Obstruction Secondary to Endometriosis. J. Clin. Gastroenterol..

[73] Singh K.K., Lessells A.M., Adam D.J., Jordan C., Miles W.F.A., MacIntyre I.M.C., Greig J.D. (2005). Presentation of Endometriosis to General Surgeons: A 10-Year Experience. Br. J. Surg..

[74] Slesser A.A.P., Sultan S., Kubba F., Sellu D.P. (2010). Acute Small Bowel Obstruction Secondary to Intestinal Endometriosis, an Elusive Condition: A Case Report. World J. Emerg. Surg..

[75] Asanza-Llorente J.A., Serrano-Egea A., López-López A., García-Aparicio M., Calderón-Duque T., Timón-Peralta J. (2013). Enterovesical Fistula and Intestinal Obstruction by Ileal Endometriosis. Rev. Esp. Enferm. Dig..

[76] Popivanov G., Stoyanova D., Fakirova A., Konakchieva M., Stefanov D., Kjossev K., Mutafchiyski V. (2020). Ileus Caused by Small Bowel, Ileocaecal and Rectal Endometriosis Misdiagnosed as Crohn’s Disease and Managed by Synchronous Ileocaecal and Rectal Resection. Ann. R. Coll. Surg. Engl..

[77] Bacalbasa N., Balescu I., Filipescu A. (2017). Ileocecal Obstruction Due to Endometriosis—A Case Report and Literature Review. In Vivo.

[78] Shetty S., Varma D. (2021). Rare Case of Ileocecal Obstruction Secondary to Endometriosis Presenting for the First Time. Cureus.

[79] Preziosi G., Cristaldi M., Angelini L. (2007). Intestinal Obstruction Secondary to Endometriosis: A Rare Case of Synchronous Bowel Localization. Surg. Oncol..

[80] Benigno L., Lisarelli L., Sortino R., Neuweiler J., Steffen T. (2020). A Rare Case of Ileocolic Intussusception Due to Severe Endometriosis. J. Surg. Case Rep..

[81] Fujimoto A., Osuga Y., Tsutsumi O., Fujii T., Okagaki R., Taketani Y. (2001). Successful Laparoscopic Treatment of Ileo-Cecal Endometriosis Producing Bowel Obstruction. J. Obstet. Gynaecol. Res..

[82] Vahdat M., Sariri E., Mehdizadeh A., Najmi Z., Shayanfar N. (2013). Colonic Obstruction as an Unusual Presentation of Endometrioma: A Case Report. Surg. Laparosc. Endosc. Percutaneous Tech..

[83] Kinder C.H. (2005). Acute Small-Bowel Obstruction Due to Endometriosis. Br. J. Surg..

[84] Nagar H.S., Tyagi A.K., Chouhan A., Mohanty S.K. (2005). Ileocaecal Endometriosis with Intestinal Obstruction. Med. J. Armed Forces India.

[85] Imasogie D.E., Agbonrofo P.I., Momoh M.I., Obaseki D.E., Obahiagbon I., Azeke A.T. (2018). Intestinal Obstruction Secondary to Cecal Endometriosis. Niger. J. Clin. Pract..

[86] Molina G.A., Ramos D.R., Yu A., Paute P.A., Llerena P.S., Alexandra Valencia S., Fonseca J.V., Morillo J.F., López S.C., Gutierrez B.M. (2019). Endometriosis Mimicking a Cecum Mass with Complete Bowel Obstruction: An Infrequent Cause of Acute Abdomen. Case Rep. Surg..

[87] Katagiri H., Lefor A.K., Nakata T., Matsuo T., Shimokawa I. (2014). Intussusception Secondary to Endometriosis of the Cecum. Int. J. Surg. Case Rep..

[88] Nozari N., Shafiei M., Sarmadi S. (2018). An Unusual Presentation of Endometriosis as an Ileocolic Intussusception with Cecal Mass: A Case Report. J. Reprod. Infertil..

[89] Alexandrino G., Lourenço L.C., Carvalho R., Sobrinho C., Horta D.V., Reis J. (2018). Endometriosis: A Rare Cause of Large Bowel Obstruction. GE Port. J. Gastroenterol..

[90] Alvarado L.E.R., Bahmad H., Mejia O., Hollembeak H., Poppiti R., Howard L., Muddasani K. (2021). Rectal Endometriosis Presenting as Toxic Megacolon. Autops. Case Rep..

[91] Katsikogiannis N., Tsaroucha A.K., Dimakis K., Sivridis E., Simopoulos C.E. (2011). Rectal Endometriosis Causing Colonic Obstruction and Concurrent Endometriosis of the Appendix: A Case Report. J. Med. Case Rep..

[92] Ono H., Honda S., Danjo Y., Nakamura K., Okabe M., Kimura T., Kawakami M., Nagashima K., Nishihara H. (2014). Rectal Obstruction Due to Endometriosis: A Case Report and Review of the Japanese Literature. Int. J. Surg. Case Rep..

[93] Mourthé De Alvim Andrade M., Batista Pimenta M., de Freitas Belezia B., Duarte T. (2008). Rectal Obstruction Due to Endometriosis. Tech. Coloproctol..

[94] Jarmin R., Idris M.A., Shaharuddin S., Nadeson S., Rashid L.M., Mustaffa W.M.W. (2006). Intestinal Obstruction Due to Rectal Endometriosis: A Surgical Enigma. Asian J. Surg..

[95] Xu Y., Xu Y., Miao L., Cao M., Xu W., Shi L. (2022). Comprehensive Surgical Treatment for Obstructive Rectal Endometriosis: A Case Report and Review of the Literature. BMC Women’s Health.

[96] Falleni M., Bauer D., Opocher E., Moneghini L., Bulfamante G.P. (2014). A rare case of transmural endometriosis in primary adenocarcinoma of the rectum. Pathologica.

[97] Insabato L., D’Armiento F.P., Tornillo L. (1994). A Rectal Endometrioma Producing Intestinal Obstruction. J. Clin. Gastroenterol..

[98] Galazis N., Arul D., Wilson J., Pisal N. (2014). Bowel endometriosis. BMJ Case Rep..

[99] Stevens K., Wasfie T., Haus C. (2021). Endometrioma Causing Near-Complete Obstruction of the Sigmoid Colon. Am. Surg..

[100] El Bakouri A., El Karouachi A., Bouali M., Khouaja A., Elhattabi K., Bensardi F., Fadil A., Karkouri M. (2021). Acute Colonic Occlusion over Endometriosis: About a Case. Int. J. Surg. Case Rep..

[101] Buldanlı M.Z., Özemir İ.A., Yener O., Dölek Y. (2020). A Rare Case of Acute Mechanical Intestinal Obstruction: Colonic Endometriosis. Ulus. Travma Acil Cerrahi Derg..

[102] Shaw A., Lund J.N., Semeraro D., Cartmill M., Reynolds J.R., Tierney G.M. (2008). Large Bowel Obstruction and Perforation Secondary to Endometriosis Complicated by a Ventriculoperitoneal Shunt. Color. Dis..

[103] Bascombe N.A., Naraynsingh V., Dan D., Harnanan D. (2013). Isolated Endometriosis Causing Sigmoid Colon Obstruction: A Case Report. Int. J. Surg. Case Rep..

[104] Baden D.N., van de Ven A., Verbeek P.C. (2015). Endometriosis with an Acute Colon Obstruction: A Case Report. J. Med. Case Rep..

[105] Sarofim M., Attwell-Heap A., Trautman J., Kwok A., Still A. (2019). Unusual Case of Acute Large Bowel Obstruction: Endometriosis Mimicking Sigmoid Malignancy. ANZ J. Surg..

[106] De Weerdt V., Bossuyt P., Peperstraete L. (2014). Colonic obstruction in a 45 year old female. Acta Gastro Enterol. Belg..

[107] Al-Qahtani H.H., Alfalah H., Al-Salamah R.A., Elshair A.A. (2015). Sigmoid Colon Endometriotic Mass. Saudi Med. J..

[108] Arafat S., Alsabek M.B., Almousa F., Kubtan M.A. (2016). Rare Manifestation of Endometriosis Causing Complete Recto-Sigmoid Obstruction: A Case Report. Int. J. Surg. Case Rep..

[109] Rana R., Sharma S., Narula H., Madhok B. (2016). A Case of Recto-Sigmoid Endometriosis Mimicking Carcinoma. SpringerPlus.

[110] Murji A., Sobel M.L. (2011). Bowel Obstruction and Pelvic Mass. Can. Med. Assoc. J..

[111] de Jong M.J.H., Mijatovic V., van Waesberghe J.H.T.M., Cuesta M.A., Hompes P.G.A. (2009). Surgical Outcome and Long-Term Follow-up after Segmental Colorectal Resection in Women with a Complete Obstruction of the Rectosigmoid Due to Endometriosis. Dig. Surg..

[112] Pramateftakis M.G., Psomas S., Kanellos D., Vrakas G., Roidos G., Makrantonakis A., Kanellos I. (2010). Large Bowel Obstruction Due to Endometriosis. Tech. Coloproctol..

[113] Lin Y.-H., Kuo L.-J., Chuang A.-Y., Cheng T.-I., Hung C.-F. (2006). Extrapelvic Endometriosis Complicated with Colonic Obstruction. J. Chin. Med. Assoc..

[114] Caselli G., Besa C., Pulgar D. (2011). Intestinal Obstruction as Manifestation of a Multifocal Colonic Endometriosis. Clin. Gastroenterol. Hepatol..

[115] Yildirim S., Nursal T.Z., Tarim A., Torer N., Bal N., Yildirim T. (2005). Colonic Obstruction Due to Rectal Endometriosis: Report of a Case. Turk. J. Gastroenterol..

[116] Tate G.T. (1963). Acute Obstruction of the Large Bowel Due to Endometriosis. Br. J. Surg..

[117] Lanitis S., Korontzi M., Karaliotas C. (2013). Acute Bowel Obstruction in a Premenopausal Woman. Gastroenterology.

[118] Eyers T., Morgan B., Bignold L. (1978). Endometriosis Of The Sigmoid Colon And Rectum. ANZ J. Surg..

[119] Sassi S., Bouassida M., Touinsi H., Mongi Mighri M., Baccari S., Chebbi F., Bouzeidi K., Sassi S. (2011). Exceptional Cause of Bowel Obstruction: Rectal Endometriosis Mimicking Carcinoma of Rectum—A Case Report. Pan. Afr. Med. J..

[120] Choi J.D.W., Yunaev M. (2019). Endometriosis of the Appendix Causing Small Bowel Obstruction in a Virgin Abdomen. BMJ Case Rep..

[121] Jeong S.J., Park J. (2020). Endoscopic Management of Benign Colonic Obstruction and Pseudo-Obstruction. Clin. Endosc..

[122] Beck D.E. (2021). Endoscopic Management of Bowel Obstruction. Clin. Colon Rectal Surg..

[123] Lemberg B., Vargo J.J. (2007). Balloon Dilation of Colonic Strictures. Am. J. Gastroenterol..

[124] Small A.J., Young-Fadok T.M., Baron T.H. (2008). Expandable Metal Stent Placement for Benign Colorectal Obstruction: Outcomes for 23 Cases. Surg. Endosc..

[125] Lee J.-M., Byeon J.-S. (2015). Colorectal Stents: Current Status. Clin. Endosc..

[126] Baron T.H. (2005). Colonic Stenting: Technique, Technology, and Outcomes for Malignant and Benign Disease. Gastrointest. Endosc. Clin. N. Am..

[127] Young S., Kennedy Burns M., DiFrancesco L., Nezhat A., Nezhat C. (2017). Diagnostic and Treatment Guidelines for Gastrointestinal and Genitourinary Endometriosis. J. Turk.-Ger. Gynecol. Assoc..

[128] Nezhat C., Li A., Falik R., Copeland D., Razavi G., Shakib A., Mihailide C., Bamford H., DiFrancesco L., Tazuke S. (2018). Bowel Endometriosis: Diagnosis and Management. Am. J. Obstet. Gynecol..

[129] Remorgida V., Ferrero S., Fulcheri E., Ragni N., Martin D.C. (2007). Bowel Endometriosis: Presentation, Diagnosis, and Treatment. Obstet. Gynecol. Surv..

[130] Egekvist A.G., Marinovskij E., Forman A., Kesmodel U.S., Riiskjaer M., Seyer-Hansen M. (2017). Conservative Approach to Rectosigmoid Endometriosis: A Cohort Study. Acta Obstet. Gynecol. Scand..

[131] Egekvist A.G., Marinovskij E., Forman A., Kesmodel U.S., Graumann O., Seyer-Hansen M. (2019). Conservative Treatment of Rectosigmoid Endometriosis: A Prospective Study. Acta Obstet. Gynecol. Scand..

[132] Mușat F., Bolocan A., Ion D., Palcău C.A., Ginghină O., Andronic O., Oacheșu I.M., Păduraru D.N. (2022). Ileal Endometriosis—A Rare Cause Of Bowel Obstruction. Rom. J. Emerg. Surg..

