# Supplementary material for: Endometriosis as an Uncommon Cause of Intestinal Obstruction—A Comprehensive Literature Review
Source: J Clin Med. 2023 Oct 6;12(19):6376. doi: 10.3390/jcm12196376 (PMC10573381; doi:10.3390/jcm12196376)
Supplement: Supplementary file 1 [file jcm-12-06376-s001.zip › jcm-2536854-supplementary.pdf]

Table S1. Selected articles and the number of cases included from each study.

	TITLE	AUTHORS	JOURNAL	PUBLICATION YEAR	COUNTRY	NUMBER OF CASES INCLUDED
1	Intestinal obstruction caused by endometriosis: Endoscopic stenting and expedited laparoscopic resection avoiding stoma. A case report and review of the literature	Calcagno P, Viti M, Cornelli A, Galli D, D'Urbano C.	Int J Surg Case Rep	2018	Italy	1
2	Small Bowel Obstruction Caused by Ileal Endometriosis with Appendiceal and Lymph Node Involvement Treated with Single-Incision Laparoscopic Surgery: A Case Report and Review of the Literature	Koyama R, Aiyama T, Yokoyama R, Nakano S.	Am J Case Rep	2021	Japan	1
3	Bowel endometriosis	Galazis N, Arul D, Wilson J, Pisal N.	BMJ Case Rep	2014	UK	1
4	Endometriosis: A Rare Cause of Large Bowel Obstruction	Alexandrino G, Lourenço LC, Carvalho R, Sobrinho C, Horta DV, Reis J.	GE Port J Gastroentero l	2018	Portugal	1
5	A case of endometriosis causing acute large bowel obstruction	Allan Z.	Int J Surg Case Rep	2018	Australia	1
6	Acute small bowel obstruction secondary to intestinal endometriosis, an elusive condition: a case report	Slesser AA, Sultan S, Kubba F, Sellu DP.	World J Emerg Surg	2010	UK	1
7	Small bowel obstruction due to an endometriotic ileal stricture with associated appendiceal endometriosis: A case report and systematic review of the literature	Sali PA, Yadav KS, Desai GS, Bhole BP, George A, Parikh SS, Mehta HS.	Int J Surg Case Rep	2016	India	1
8	Acute Small Bowel Obstruction and Small Bowel Perforation as a Clinical Debut of Intestinal Endometriosis: A Report of Four Cases and Review of the Literature	Torralba-Morón A, Urbanowicz M, Ibarrola-De Andres C, Lopez-Alonso G, Colina-Ruizdelgado F, Guerra-Vales JM.	Intern Med	2016	Spain	2
9	Endometrioma Causing Near-Complete Obstruction of the Sigmoid Colon	Stevens K, Wasfie T, Haus C.	Am Surg	2021	USA	1
10	Acute small bowel obstruction caused by endometriosis: a case report and review of the literature	De Ceglie A, Bilardi C, Blanchi S, Picasso M, Di Muzio M, Trimarchi A, Conio M.	World J Gastroentero l	2008	Italy	1
11	Endometriosis as a rare cause of small bowel obstruction	Lam K, Lang E.	ANZ J Surg	2020	Australia	1
12	Small bowel obstruction caused by appendiceal and ileal endometriosis: a case report	Kobayashi K, Yamadera M, Takeo H, Murayama M.	J Surg Case Rep	2022	Japan	1
13	Rectal endometriosis presenting as toxic megacolon	Alvarado LER, Bahmad H, Mejia O, Hollembeak H, Poppiti R, Howard L, Muddasani K.	Autops Case Rep	2021	USA	1
14	Intestinal obstruction secondary to cecal endometriosis	Imasogie DE, Agbonrofo PI, Momoh MI, Obaseki DE, Obahiagbon I, Azeke AT.	Niger J Clin Pract	2018	Nigeria	1
15	Acute colonic occlusion over endometriosis: About a case	El Bakouri A, El Karouachi A, Bouali M, Khouaja A, Elhattabi K, Bensardi F, Fadil A, Karkouri M.	Int J Surg Case Rep	2021	Morocco	1
16	A rare case of acute mechanical intestinal obstruction: Colonic endometriosis	Buldanlı MZ, Özemir İA, Yener O, Dölek Y.	Ulus Travma Acil Cerrahi Derg	2020	Turkey	1
17	A case report of endometriosis presenting as an acute small bowel obstruction	Chan DL, Chua D, Ravindran P, Perez Cerdeira M, Mor I.	Int J Surg Case Rep	2017	Australia	1

18	Ileocecal endometriosis: diagnosis and management	López Carrasco A, Hernández Gutiérrez A, Hidalgo Gutiérrez PA, Rodríguez González R, Marijuán Martín JL, Zapardiel I, de Santiago García J.	Taiwan J Obstet Gynecol	2017	Spain	1
19	A Rare Case of Endometriosis of the Small Bowel	Zepeda MR, Win SK.	Case Rep Pathol	2021	USA	1
20	Large bowel obstruction and perforation secondary to endometriosis complicated by a ventriculoperitoneal shunt	Shaw A, Lund JN, Semeraro D, Cartmill M, Reynolds JR, Tierney GM.	Colorectal Dis	2008	UK	1
21	Anasarca and small bowel obstruction secondary to endometriosis	Mussa FF, Younes Z, Tihan T, Lacy BE.	J Clin Gastroentero l	2001	USA	1
22	Rectal endometriosis causing colonic obstruction and concurrent endometriosis of the appendix: a case report	Katsikogiannis N, Tsaroucha A, Dimakis K, Sivridis E, Simopoulos C.	J Med Case Rep	2011	Greece	1
23	Ileocolonic intussusception due to severe endometriosis	Morales-Morales CA, Morales-Flores LF, Gonzalez-Urquijo M, Suárez-Márquez E, Zambrano-Lara M, Baca-Arzaga AA, Tijerina-Gómez LO.	Clin J Gastroentero l	2021	Mexico	1
24	Enterovesical fistula and intestinal obstruction by ileal endometriosis	Asanza-Llorente JA, Serrano-Egea A, López-López A, García-Aparicio M, Calderón-Duque T, Timón-Peralta J.	Rev Esp Enferm Dig	2013	Spain	1
25	Postmenopausal intestinal obstructive endometriosis: case report and review of the literature	Popoutchi P, dos Reis Lemos CR, Silva JC, Nogueira AA, Feres O, Ribeiro da Rocha JJ.	Sao Paulo Med J	2008	Brazil	1
26	Ileus caused by small bowel, ileocaecal and rectal endometriosis misdiagnosed as Crohn's disease and managed by synchronous ileocaecal and rectal resection	Popivanov G, Stoyanova D, Fakirova A, Konakchieva M, Stefanov D, Kjossev K, Mutafchiyski V.	Ann R Coll Surg Engl	2020	Bulgaria	1
27	Endometriosis Mimicking a Cecum Mass with Complete Bowel Obstruction: An Infrequent Cause of Acute Abdomen	Molina GA, Ramos DR, Yu A, Paute PA, Llerena PS, Alexandra Valencia S, Fonseca JV, Morillo JF, López SC, Gutierrez BM.	Case Rep Surg	2019	Ecuador	1
28	Sigmoid endometriosis in a post-menopausal woman leading to acute large bowel obstruction: A case report	Bidarmaghz B, Shekhar A, Hendahewa R.	Int J Surg Case Rep	2016	Australia	1
29	Intussusception secondary to endometriosis of the cecum	Katagiri H, Lefor AK, Nakata T, Matsuo T, Shimokawa I.	Int J Surg Case Rep	2014	Japan	1
30	Ileocecal Obstruction Due to Endometriosis - A Case Report and Literature Review	Bacalbasa N, Balescu I, Filipescu A.	In Vivo	2017	Romania	1
31	Rare Case of Ileocecal Obstruction Secondary to Endometriosis Presenting for the First Time	Shetty S, Varma D.	Cureus	2021	Cayman Island+Ind ia	1
32	A case of ileus caused by ileal endometriosis with lymph node involvement	Arata R, Takakura Y, Ikeda S, Itamoto T.	Int J Surg Case Rep	2019	Japan	1
33	Ileal obstruction caused by transmural endometriosis in a patient with simultaneous C. difficile colitis and Influenza AH1N1. Case report	Santos-Manzur A, Valdez-Bocanegra DR, Ornelas-Flores MC, Pineda-Díaz J, Stoopen-Margain E.	Int J Surg Case Rep	2020	Mexico	1

34	Endometriosis of the appendix causing small bowel obstruction in a virgin abdomen	Choi JDW, Yunaev M.	BMJ Case Rep	2019	Australia	1
35	Bowel obstruction secondary to deep infiltrating endometriosis of the ileum	Ávila Vergara MA, Sánchez Carrillo V, Peraza Garay F.	Rev Esp Enferm Dig	2018	Mexico	1
36	Rare manifestation of endometriosis causing complete recto-sigmoid obstruction: A case report	Arafat S, Alsabek MB, Almousa F, Kubtan MA.	Int J Surg Case Rep	2016	Syria	1
37	A case of recto-sigmoid endometriosis mimicking carcinoma	Rana R, Sharma S, Narula H, Madhok B.	Springerplus	2016	UK	1
38	An Unusual Cause of Large Bowel Obstruction in a Patient With Ulcerative Colitis	Moktan VP, Koop AH, Olson MT, Lewis MD, Gomez V, Farraye FA.	ACG Case Rep J	2021	USA	1
39	Metachronic malignant transformation of small bowel and rectal endometriosis in the same patient	Marchena-Gomez J, Conde-Martel A, Hemmersbach-Miller M, Alonso-Fernandez A.	World J Surg Oncol	2006	Spain	1
40	Intestinal obstruction secondary to endometriosis: a rare case of synchronous bowel localization	Preziosi G, Cristaldi M, Angelini L.	Surg Oncol	2007	UK	1
41	Anastomotic Leakage in a Patient with Acute Intestinal Obstruction Secondary to Appendiceal and Ileal Endometriosis: A Case Report	Arer IM, Yabanoglu H, Hasbay B.	J Clin Diagn Res	2016	Turkey	1
42	Endometriosis: a rare cause of small bowel obstruction	Khwaja SA, Zakaria R, Carneiro HA, Khwaja HA.	BMJ Case Rep	2012	UK	1
43	Beyond borders: A case report of small bowel obstruction secondary to undiagnosed florid endometriosis	Dhannoon A, Bajwa A, Kunna M, Canney A, Nugent E.	Int J Surg Case Rep	2022	Ireland	1
44	Endometriosis of the terminal ileum: a diagnostic dilemma	Karaman K, Pala EE, Bayol U, Akman O, Olmez M, Unluoglu S, Ozturk S.	Case Rep Pathol	2012	Turkey	1
45	Free large sized intra-abdominal endometrioma in a postmenopausal woman: a case report	Naem A, Shamandi A, Al-Shiekh A, Alsaid B.	BMC Womens Health	2020	Syria	1
46	Small bowel obstruction caused by endometriosis in a postmenopausal woman	Izuishi K, Sano T, Shiota A, Mori H, Ebara K.	Asian J Endosc Surg	2015	Japan	1
47	Acute endometrial bowel obstruction-A rare indication for colonic stenting	Whelton C, Bhowmick A.	Int J Surg Case Rep	2013	UK	1
48	A rare case of ileus caused by ileum endometriosis	Bratu D, Chicea R, Ciprian T, Beli L, Dan S, Mihetiu A, Adrian B.	Int J Surg Case Rep	2016	Romania	1
49	Bowel obstruction and pelvic mass	Murji A, Sobel ML.	CMAJ	2011	Canada	1
50	Irritable bowel syndrome or endometriosis, or both?	Lea R, Whorwell PJ.	Eur J Gastroenterol Hepatol	2003	UK	1
51	Laparoscopic Triple Segmental Bowel Resection for Endometriosis Revealed by Rectal Obstruction during Infertility Treatment	Nagakari K, Azuma D, Takehara K, Ohuchi M, Ishizaki Y, Sakamoto K.	Case Rep Gastroenterol	2022	Japan	1
52	Isolated endometriosis causing sigmoid colon obstruction: A case report	Bascombe NA, Naraynsingh V, Dan D, Harnanan D.	Int J Surg Case Rep	2013	Trinidad and Tobago	1
53	Surgical outcome and long-term follow-up after segmental colorectal resection in women with a complete obstruction of the rectosigmoid due to endometriosis	de Jong MJ, Mijatovic V, van Waesberghe JH, Cuesta MA, Hompes PG.	Dig Surg	2009	Netherlands	5
54	Endometriosis with an acute colon obstruction: a case report	Baden DN, van de Ven A, Verbeek PC.	J Med Case Rep	2015	Netherlands	1
55	An Unusual Presentation of Endometriosis as an Ileocolic	Nozari N, Shafiei M, Sarmadi S.	J Reprod Infertil	2018	Iran	1

	Intussusception with Cecal Mass: A Case Report					
56	A rare case of ileocolic intussusception due to severe endometriosis	Benigno L, Lisarelli L, Sortino R, Neuweiler J, Steffen T.	J Surg Case Rep	2020	Switzerlan d	1
57	Intestinal endometriosis masquerading as common digestive disorders	Shah M, Tager D, Feller E.	Arch Intern Med	1995	USA	2
58	Rectal obstruction due to endometriosis: A case report and review of the Japanese literature	Ono H, Honda S, Danjo Y, Nakamura K, Okabe M, Kimura T, Kawakami M, Nagashima K, Nishihara H.	Int J Surg Case Rep	2014	Japan	1
59	Rectal obstruction due to endometriosis	Mourthé de Alvim Andrade M, Batista Pimenta M, de Freitas Belezia B, Duarte T.	Tech Coloproctol	2008	Brazil	2
60	Exceptional cause of bowel obstruction: rectal endometriosis mimicking carcinoma of rectum--a case report	Sassi S, Bouassida M, Touinsi H, Mongi Mighri M, Baccari S, Chebbi F, Bouzeidi K, Sassi S.	Pan Afr Med J	2011	Tunisia	1
61	Unusual case of acute large bowel obstruction: endometriosis mimicking sigmoid malignancy	Sarofim M, Attwell-Heap A, Trautman J, Kwok A, Still A.	ANZ J Surg	2019	Australia	1
62	Terminal ilial intussusception in an adult due to endometriosis	Ranaweera RK, Gamage SM, Ubayawansa DH, Kumara MM.	BMC Res Notes	2016	Sri Lanka	1
63	Laparoscopic management of a small bowel obstruction caused by an endometriotic focus	Laiz Díez B, García Muñoz-Najar A, Durán Poveda M.	Rev Esp Enferm Dig	2019	Spain	1
64	Large bowel obstruction due to endometriosis	Pramateftakis MG, Psomas S, Kanellos D, Vrakas G, Roidos G, Makrantonakis A, Kanellos I.	Tech Coloproctol	2010	Greece	3
65	Endometriosis masquerading as Crohn's disease in a patient with acute small bowel obstruction	Dong C, Ngu WS, Wakefield SE.	BMJ Case Rep	2015	UK	1
66	Stromal endometriosis of the intestine: an elusive presentation with a review of the literature: a case report	Upreti S, Bansal R, Upreti S, Mathur S.	J Clin Diagn Res	2013	India	1
67	Acute small bowel obstruction secondary to ileal endometriosis: report of a case	Ridha JR, Cassaro S.	Surg Today	2003	USA	1
69	Acute small bowel obstruction due to endometriosis	POOLE RW.	Can Med Assoc J	1961	Canada	1
70	Intestinal obstruction due to rectal endometriosis: a surgical enigma	Jarmin R, Idris MA, Shaharuddin S, Nadeson S, Rashid LM, Mustaffa WM.	Asian J Surg	2006	Malaysia	1
71	Distal ileal endometriosis as a cause of ileus: a case report	Gregorić P, Doklestić K, Pandurović M, Radenković D, Karadzić B, Raspopović M, Micev M, Ivancević N, Sijacki A, Bajec D.	Srp Arh Celok Lek	2012	Serbia	1
72	Comprehensive surgical treatment for obstructive rectal endometriosis: a case report and review of the literature	Xu Y, Xu Y, Miao L, Cao M, Xu W, Shi L.	BMC Womens Health	2022	China	1
73	Small bowel obstruction caused by endometriosis	Attar A, Lagorce C.	Clin Gastroentero l Hepatol	2007	France	1
74	Extrapelvic endometriosis complicated with colonic obstruction	Lin YH, Kuo LJ, Chuang AY, Cheng TI, Hung CF.	J Chin Med Assoc	2006	Taiwan	1
75	Intestinal obstruction as manifestation of a multifocal colonic endometriosis	Caselli G, Besa C, Pulgar D.	Clin Gastroentero l Hepatol	2011	Chile	1
76	Colonic obstruction in a 45 year old female	De Weerdt V, Bossuyt P, Peperstraete L.	Acta Gastroentero l Belg	2014	Belgium	1

77	Ileocolic intussusception due to endometriosis	Koutsourelakis I, Markakis H, Koulas S, Mparmpantonakis N, Perraki E, Christodoulou K.	JSLS	2007	Greece	1
78	Colonic obstruction due to rectal endometriosis: report of a case	Yildirim S, Nursal TZ, Tarim A, Torer N, Bal N, Yildirim T.	Turk J Gastroenterol	2005	Turkey	1
79	Colonic obstruction caused by endometriosis solved with a colonic stent as a bridge to surgery	Navajas-Laboa M, Orive-Calzada A, Landaluce A, Zabalza-Estevez I, Larena JA, Arévalo-Serna JA, Bridet L, López-López M, Torres-Burgos S, Bernal-Martínez A, Cabriada-Nuño JL.	Arab J Gastroenterol	2015	Spain	1
80	Ileal obstruction due to endometriosis, with associated appendiceal involvement: A unique and elusive situation	Rancaño RS, Choho KK, Morales MD, Bueno VMS, Soto MDME, Santacruz YR, Guillén RA.	Gastroenterol Hepatol	2022	Spain	1
81	Successful laparoscopic treatment of ileo-cecal endometriosis producing bowel obstruction	Fujimoto A, Osuga Y, Tsutsumi O, Fujii T, Okagaki R, Taketani Y.	J Obstet Gynaecol Res	2001	Japan	1
82	Sigmoid colon endometriotic mass. A rare cause of complete large bowel obstruction	Al-Qahtani HH, Alfalah H, Al-Salamah RA, Elshair AA.	Saudi Med J	2015	Kingdom of Saudi Arabia	1
83	Small bowel obstruction secondary to ileal endometriosis: multisection computer tomography evaluation	Fernández-Rey CL, Alvarez-González SA, Díaz-Solís P, Blanco-González A, Costilla-García S.	Rev Esp Enferm Dig	2009	Spain	1
84	Presentation of endometriosis to general surgeons: a 10-year experience	Singh KK, Lessells AM, Adam DJ, Jordan C, Miles WF, Macintyre IM, Greig JD.	Br J Surg	1995	UK	2
85	ACUTE OBSTRUCTION OF THE LARGE BOWEL DUE TO ENDOMETRIOSIS	TATE GT.	Br J Surg	1963	UK	1
86	Endometriosis of the sigmoid colon and rectum	Eyers T, Morgan B, Bignold L.	Aust N Z J Surg	1978	Australia	1
87	Colonic obstruction as an unusual presentation of endometrioma: a case report	Vahdat M, Sariri E, Mehdizadeh A, Najmi Z, Shayanfar N.	Surg Laparosc Endosc Percutan Tech	2013	Iran	1
88	A rare case of transmural endometriosis in primary adenocarcinoma of the rectum	Falleni M, Bauer D, Opocher E, Moneghini L, Bulfamante GP.	Pathologica	2014	Italy	1
89	Invasive endometriosis of the terminal ileum: a cause of small bowel obstruction of obscure origin	Harty RF, Kaude JV.	South Med J	1983	USA	1
90	Acute bowel obstruction in a premenopausal woman	Lanitis S, Korontzi M, Karaliotas C.	Gastroenterology	2013	Greece	1
91	Acute small bowel obstruction due to endometriosis	KINDER CH.	Br J Surg	1954	UK	1
92	Endometriosis as a cause of intestinal obstruction; a report of two cases	COLLINS PG.	Postgrad Med J	1957	UK	2
93	A rectal endometrioma producing intestinal obstruction	Insabato L, D'Armiento FP, Tornillo L.	J Clin Gastroenterol	1994	Italy	1
94	Sigmoid endometriosis in a postmenopausal woman	Deval B, Rafii A, Felce Dachez M, Kermanash R, Levardon M.	Am J Obstet Gynecol	2002	France	1
95	Ileocaecal Endometriosis with Intestinal Obstruction	Nagar HS, Tyagi AK, Chouhan A, Mohanty SK.	Med J Armed Forces India	2005	India	1
96	Bowel occlusion in an infertile woman with documented deep endometriosis	Quicray M, Darwish B, Bridoux V, Roman H.	Gynecol Obstet Fertil.	2016	France	1

	of the sigmoid colon: Why was it not unexpected?					
97	Extraluminal bowel obstruction by endometrioid adenocarcinoma 34 years post-hysterectomy: risks of unopposed oestrogen therapy	Wang TT, Jabbour RJ, Girling JC, McDonald PJ.	J R Soc Med	2011	UK	1
						107
